# Diné (Navajo) Traditional Knowledge Holders’ Perspective of COVID-19

**DOI:** 10.3390/ijerph20043728

**Published:** 2023-02-20

**Authors:** Carmella B. Kahn, DeeDee James, Shawndeena George, Tressica Johnson, Michelle Kahn-John, Nicolette I. Teufel-Shone, Chassity Begay, Marissa Tutt, Mark C. Bauer

**Affiliations:** 1 College of Population Health, University of New Mexico, Albuquerque, NM 87131, USA; 2 Center for Health Equity and Research, Northern Arizona University, Flagstaff, AZ 86011, USA; 3 Public Health Program, Diné College, Shiprock, NM 87420, USA; 4 School of Nursing, Johns Hopkins University, Baltimore, MD 21205, USA

**Keywords:** traditional knowledge holder, medicine men, medicine women, practitioner, Diné, Navajo, COVID-19 pandemic, Hózhó Resilience Model, American Indian

## Abstract

From the start of the COVID-19 pandemic on the Navajo Nation, Diné (Navajo) traditional knowledge holders (TKHs), such as medicine men and women and traditional practitioners, contributed their services and healing practices. Although TKHs are not always fully acknowledged in the western health care system, they have an established role to protect and promote the health of Diné people. To date, their roles in mitigating the COVID-19 pandemic have not been fully explored. The purpose of this research was to understand the social and cultural contexts of the COVID-19 pandemic and vaccines based on the roles and perspectives of Diné TKHs. A multi-investigator consensus analysis was conducted by six American Indian researchers using interviews with TKHs collected between December 2021–January 2022. The Hózhó Resilience Model was used as a framework to analyze the data using four parent themes: COVID-19, harmony and relationships, spirituality, and respect for self and discipline. These parent themes were further organized into promoters and/or barriers for 12 sub-themes that emerged from the data, such as traditional knowledge, Diné identity, and vaccine. Overall, the analysis showed key factors that could be applied in pandemic planning and public health mitigation efforts based on the cultural perspective of TKHs.

## 1. Introduction

The coronavirus disease 2019 (COVID-19) pandemic has shed light on significant inequities within the American Indian (AI) health care system that have existed in the United States (US) for decades. The heaviest burden of COVID-19 was predominately among African American, Hispanic, and AI communities [1]. The Navajo Nation (NN) is the largest AI reservation in the US and encompasses over 27,000 square miles across three states, Arizona, Utah, and New Mexico [2]. According to the NN Office of the Controller, the Navajo (Diné) tribe is also the largest in the US, with 399,494 enrolled members [3]. In this paper, the word Navajo will be used interchangeably with Diné, which is how Navajo identify themselves in their native language. The NN has long experienced barriers in the health care system due to underfunding and infrastructural deficiencies coupled with historical trauma from centuries of forced relocation and broken federal treaties [4]. Despite strong public health efforts, the COVID-19 pandemic swept across the country. The pre-existing NN psychosocial disadvantages resulted in disproportionate impacts and one of the highest per capita rates of COVID-19 in the US [5].

Within two months of the first COVID-19 case on the NN in March 2020, the NN became a hotspot and experienced the highest per capita infection rate of 2304.41 cases per 100,000 people and surpassed all U.S. states, including New York and New Jersey [6,7]. Despite precautionary measures, American Indian and Alaska Native (AI/AN) persons are 1.6 times more likely to contract COVID-19, 2.7 times more likely to be hospitalized because of COVID-19, and 2.1 times more likely to die from COVID-19 complications compared to non-Hispanic White persons [8]. By May 2020, there were 3245 confirmed COVID-19 cases across the NN and 103 confirmed deaths among Diné people [9]. NN leaders and public health officials continue efforts to mitigate viral spread and refine policies to protect and preserve the Diné way of life.

The environmental conditions in which Diné people live significantly impact their health and wellbeing. Many households across the reservation lack basic needs such as electricity and running water. More than 40% of Diné families lack indoor plumbing and must rely on hauling water for household consumption [10]. Only 13 grocery stores and 12 medical facilities serve the NN, causing Diné families to commute off the reservation to buy necessities and personal protective equipment (PPE) [2,11]. COVID-19 revealed many inadequacies in the national US health care system that impacted AI health care, such as infrastructure shortfalls, accurate data collection and reporting limitations, distribution and access to COVID-19 screening tests, inconsistencies in disseminating factual real-time information, and insufficient access to resources such as PPE [12].

Traditional knowledge holders (TKHs), such as medicine men and women and specialty practitioners, maintain an essential role as keepers of traditional teachings on how to sustain wellness and balance, and their teachings were critical in helping the Diné overcome COVID-19 related hardships. During the pandemic, some TKHs and the NN president, Jonathan Nez, were vocal in sharing relevant traditional teachings about COVID-19 and referred to it as “a monster” [13]. The NN Department of Health (NNDOH) applied a literal Diné translation of COVID-19 and named it Dikos Ntsaaígíí-19 [9].

### 1.1. Underlying Factors Influencing COVID-19 and Vaccination

The social determinants of health have influenced high rates of COVID-19, including vaccination rates. Wang [14] identified four specific social factors that heavily influenced the NN:

1. The prevalence of underlying chronic diseases.
2. The lack of institutional resilience to changes and psychosocial stresses.
3. The lack of trust between the federal government and tribal government.
4. The lack of social trust.

These factors, along with the already sluggish tribal economics and the underfunded tribal health care system, offer insight on what contributed to the high rates of COVID-19 on the NN. More research is needed to understand the health impacts of these underlying social factors.

According to the Centers for Disease Control and Prevention (CDC) [8], which provides vaccination data categorized by race and ethnic group, non-Hispanic AI/ANs have led the highest rates of receiving the first dose and complete vaccination rates in the US. A poll of approximately 2000 AI/ANs found that emphasizing the preservation of culture and language in public health mitigation efforts increased the support for vaccines [15]. A large percentage of Diné adults (69.8%) have outpaced the US national vaccine rate, indicating a large portion of Diné adults had received the vaccine [9]. However, AI/AN people have also expressed concerns and distrust with vaccine safety that is rooted in the history of negative experiences such as nonconsensual sterilization and discrimination within the health care systems in the US [16].

When the NN government decided to participate in the Pfizer-BioNTech COVID-19 vaccination trial, tribal members expressed suspicion, skepticism, and safety concerns, including whether they were being used as “guinea pigs” [17]. Despite assurances from Dr. Anthony Fauci, the country’s top infectious disease expert, there was distrust among the Diné who referred to past injustices and historical events, such as smallpox, as reasons to not support the vaccine trial [17].

### 1.2. Roles of Diné Traditional Knowledge Holders

AI/AN communities continue to rely on health services provided by TKHs and, if needed, mainstream health care providers. The Indian Health Service (IHS) serves 2.56 million AI/AN people nationwide [18]. The federal government promised the delivery of health care services to AI/ANs as part of the various treaties the federal government signed in exchange for lands ceded by the tribes [19,20]. The Navajo Area IHS employs approximately 3500 physicians, nurses, dentists, and other providers [21]. The NN uses an intercultural health approach, which is a complementary system where both allopathic and Indigenous health resources are available [22]. This system is inclusive of the cultural worldviews and healing practices and can meet patients’ health care needs through mutual respect and a shared understanding of patient care [23]. An exemplar of an integrated model of care is the Chinle IHS hospital in northern Arizona, where many residents continue to adhere to Diné traditions such as traditional healing resources. The Chinle Comprehensive Health Care Facility (CCHCF) implemented a program known as the Office of Native Medicine (ONM) to provide culturally appropriate patient-centered care by adding a team of traditional Diné practitioners to work alongside physicians and other providers [24].

Diné people may seek care from Diné TKHs to promote wellness and balance when suffering from physical, psychological, or spiritually related health symptoms and integrate Diné teachings and values to enhance their wellbeing [25]. Diné medicine men and women, also known as hataałii (chanters) in the Diné language, understand the importance of their role in the community and their influence on their patients and the community’s health. Younger generations of Diné people often venerate hataałii for their guidance and cultural wisdom. Through ceremonies and prayers that promote holistic health and wellbeing, hataałii play an influential role in establishing cultural norms and they are key players in the integration of Diné ceremonial practices and western medicine [25]. An existing organization called the Diné Hataałii Association (DHA) was developed to preserve, protect, and promote these cultural teachings. The DHA was established in the early 1970s and is comprised of hataałii, chanters, diagnosticians, herbalists, and traditional wisdom keepers. Members of the DHA are keepers of ceremonial practices on the NN, and continue to protect, promote, and preserve the Diné cultural wisdom, Diné language, and share and offer prayers for the health and wellbeing of their relatives across the NN [26].

The main goal for Diné TKHs is to empower the patient to work towards restoring balance and harmony of mind, body, and spirit [27]. TKHs can communicate with Diné deities or Holy People when conducting different ceremonies. Diné practitioners are particularly skilled at diagnosing illnesses using various techniques including hand trembling, star gazing, listening, and using crystals [28,29]. A cultural intervention or diagnosis focuses on why the illness occurred and what the patient can do to feel empowered to restore balance within him or herself.

### 1.3. Hózhó Resilience Model

**Figure 1:**
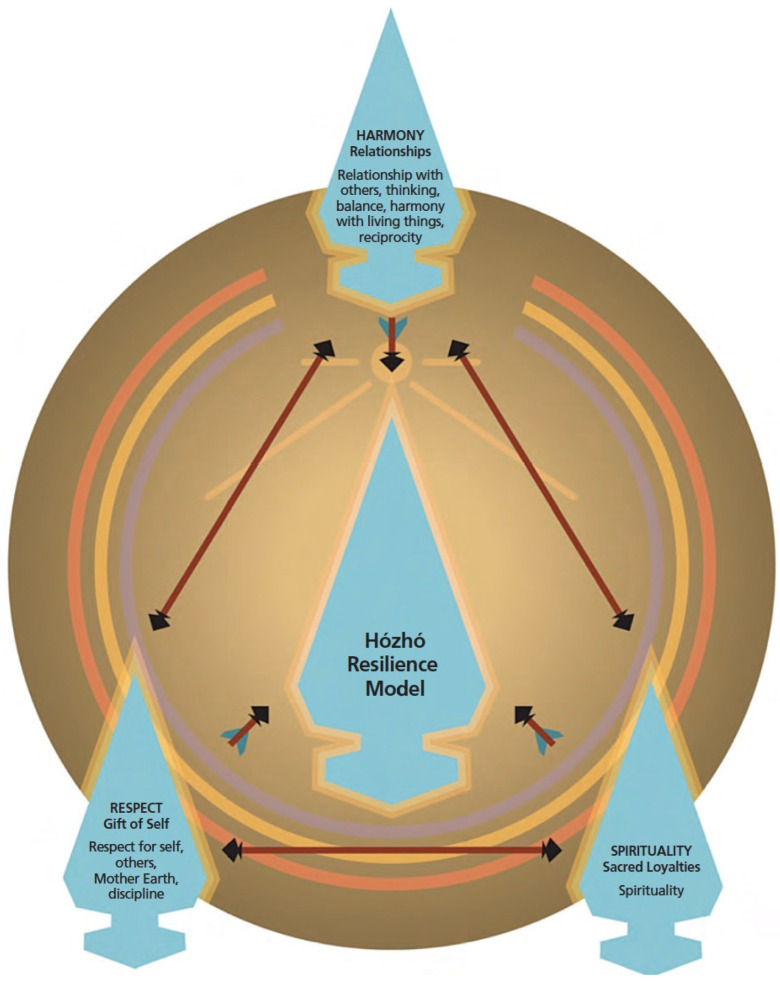
Constructs of the Hózhó Resilience Model [31].

The Hózhó Resilience Model (HRM) (Figure 1) captures the health sustaining and empowering cultural Lifeway attributes (respect, reciprocity, relationships, discipline, thinking, positivity, love, spirituality, and happiness) of the Diné [30,31]. Hózhó is a term in the Diné language that describes beauty, happiness, peace, calm, positivity, wellness, balance, and harmony within the self, others, spirit, the living world, nature, and the universe [32]. The Diné Hózhó teachings emphasize practicing and embodying these attributes as mechanisms of self-care, empowering oneself to be strong, protected, and happy as they navigate their life path while maintaining respect for others and the universe, thereby demonstrating engagement and personal responsibility in the process of creating balance, wellbeing, and harmony amongst all within the universe.

The teachings of Hózhó underscore the importance of self-awareness, individual responsibility, care of self, and generous care of others, Mother Earth, and the Universe [32]. These ageless attributes are especially relevant today as public health efforts emphasize the importance of inclusivity and equity for inhabitants of Mother Earth during the COVID-19 pandemic. The teachings also recognize that individuals are ultimately responsible for transforming their own health. The HRM emphasizes that individuals are empowered by their Diné culture and modeling attributes of Hózhó can promote physical, mental, emotional, and spiritual wellbeing.

### 1.4. Including Traditional Knowledge Holders in Research

The inclusion of TKHs in research is important because they carry diverse forms of traditional knowledge unique to each knowledge holder [33]. Much of the knowledge shared by TKHs is culturally centered on ancestral teachings and utilizes storytelling and ceremonies to understand and interpret why certain illnesses occur. Indigenous researchers value traditional knowledge and respect its origins within oral history and respect and view it as valid even within western contexts. Traditional knowledge is unique to each tribe and is not derived from a western scientific perspective [33]. Traditional stories and knowledge contain information about changes and adaptations that Diné people carry from the past that enable them to thrive for generations to come [25]. The knowledge shared by Diné TKHs is relevant for AI/AN and non-AI/AN communities. To improve whole-person wellness and promote wellbeing, Diné teachings need to be included in research. The purpose of this research is to understand the role and perspectives of TKHs, specifically within the context of cultural and social promoters and barriers of understanding the COVID-19 pandemic and vaccine uptake, and to inform and guide future public health pandemic related mitigation efforts.

## 2. Materials and Methods

TKH interviews were conducted collaboratively between Diné College (DC) and Northern Arizona University (NAU) through a supplemental project under the Navajo Native American Research Center for Health (NARCH) grant, funded by the National Institutes of Health (NIH). The project was approved by the NN Human Research Review Board. The DC team consisted of faculty and staff from the public health program, Diné Policy Institute, and a faculty member from Navajo Technical University. The NAU team included faculty and staff who are part of the Center for Health Equity and Research. Two of the DC team members completed the interviews with the help of two DC public health students who were hired as research assistants. All interviewers had training in administering interviews and self-identified as Diné.

### 2.1. Interview Guide

The interview guide was developed by the DC and NAU research partners and consisted of 13 questions. The open-ended questions ranged from asking about perspectives on COVID-19 and the vaccines, how medicine people identify COVID-19, and for advice for students on how to talk to family or friends about the vaccine. Examples of questions included: (1) How do Diné hataałii (medicine men and women) and practitioners feel about the vaccine? Based on Diné teachings, is it ok for Diné to get the COVID-19 vaccine? (2) How did Diné hataałii and practitioners educate the Navajo community regarding the traditional perspective of COVID-19? (3) What do you think about organizations like schools or tribal departments requiring vaccines? The interview guide was emailed to the participants before their scheduled interviews if they had access to email.

### 2.2. Recruitment and Sampling

Purposive sampling was used to recruit Diné TKHs (medicine men and women, and specialty practitioners). Diné TKHs are highly respected and contacting or locating a TKH can sometimes be challenging. A member of the DC team, also a trusted TKH, had contact information for other TKHs and led the recruitment efforts. A list was generated by the DC TKH team and potential participants were called and screened for eligibility. Per Diné cultural protocol, the lead interviewer began the recruitment process by visiting with the TKH to share greetings and clans, followed with a long explanation (in English and Diné) of the consent form and purpose of the project. Consent was obtained for interested participants available for interview between December 2021 to January 2022. Each recruitment discussion was conducted in the Diné language, lasted 1 h and included cultural introductions, explanation of the project and determination of availability to participate.

Fifteen (n = 15) Diné adult TKHs, all living on the NN consented to participate. The lead interviewer was fluent in the Diné language, and most of the interviews were conducted using the Diné language while others combined the use of both Diné and English language. All four interviewers were Diné, with varying degrees of fluency in the Diné language. Fluent interviewers interviewed participants who only spoke Diné. Less fluent speakers interviewed participants who spoke both English and Diné and when required, a Diné translator was requested to assist. Data collection interviews took place face-to-face, at the participant’s home or through Zoom^TM^, and lasted between 1 h to 1.5 h. Because of the cultural greeting and departure protocols, and the lengthy discussion and explanations required at recruitment and during the actual interview, the contact time with each TKH was between 3.5 to 5 h.

Each interview was audio recorded with a handheld digital recorder or via the Zoom^TM^ video and audio recording platform. The Zoom^TM^ cameras were turned off and the audio was downloaded and saved after each session. Each participant received a culturally appropriate $300.00 stipend for his/her time. The research team recognized the importance of a stipend amount that fairly compensated the TKHs for their time and knowledge. The TKHs are acknowledged as respected healing professionals in their communities who hold protected, coveted, and sacred knowledge. The research team agreed on the compensation amount for each TKH, and further justified the amount as a gesture of respect for their time, energy, and exchange of knowledge and insights that are usually keep within the circles of TKHs. Audio recorded data were transcribed by a team of five that included two Diné who were expert translators in the Diné language.

### 2.3. Data Analysis

Fifteen (n = 15) total interviews were conducted; however, only 12 (n = 12) adult interviews (11 males and one female) were included in the final analysis. There were no concerns of having only one female participant as this ratio of 11 males and 1 female is consistent with the community demographics of TKHs (typically more males than females are practicing TKHs). Because the interviews were conducted in the Diné language, translation and transcription process required extended periods of time to complete; therefore, three interviews were not included in the final analysis as they had not yet been transcribed. The research was funded by a one-year supplementary NIH grant and all research processes (including data analysis) were on a stringent timeline to meet project objectives.

The analysis was conducted by a team of six AI researchers from DC and NAU; five who self-identified as Diné. One researcher was a DC public health faculty, four other researchers were NAU public health professionals, and one researcher was an NAU graduate student in the public health program. The team used a multi-investigator consensus method borrowed from Teufel-Shone [34] that is based on Patton’s analysis methods to review qualitative data and identify content, patterns, and themes [35].

The HRM was used as a framework to guide the analysis [31]. The three main constructs, harmony/relationships, spirituality, and respect for self [discipline], were used as parent themes. Harmony was expressed as remaining positive in thought, thinking, balance, harmony with living things, and reciprocity. Respect included extending respect to self and others while exhibiting self-respect through acts of self-discipline. Spirituality included acts that honored spirit and maintaining positivity and harmony through prayer and ceremony. During the consensus analysis, sub-themes and patterns were identified and placed under the three parent themes from the HRM including an additional parent theme for COVID-19.

The six investigators split into two teams of three and each team was responsible for reviewing six interviews (combined total of 12) that were transcribed into English. All team members independently read the six transcripts assigned to their team and analyzed the content based on the four a priori parent themes. The investigators reviewed the interview transcripts and identified recurring or key concepts, patterns, and themes and organized them using a pre-designed table with the four parent themes. Each team then convened separately during weekly meetings to discuss the content analysis process, themes and achieved consensus on the identified patterns and themes. Upon completion of analysis by each team, the two teams came together for a two-day meeting and discussed their findings and reached consensus on organizing the identified themes and sub-themes within the original four parent theme categories.

## 3. Results

The research team identified 12 sub-themes under the four parent themes of COVID-19, harmony/relationships, spirituality, and respect for self/discipline. Specific promoters and/or barriers relevant to each sub-theme, are listed under each parent theme category. See sub-themes alongside identified barrier and protector patterns listed under the COVID-19 parent category in Table 1. The next section offers details on each of the identified sub-themes and barrier/protector patterns identified for each of the four parent theme categories.

### 3.1. COVID-19

#### 3.1.1. Traditional Lens towards COVID-19

Promoters for traditional lens towards COVID-19—TKHs voiced their perspectives from a traditional lens, and shared insights about the pandemic and the vaccination. Among the responses, the main patterns included the importance of creation stories and discussion on identifying a name for COVID-19 in the Diné language. A TKH shared one story, *“So I know a lot of these vaccines are made from different herbs to help us fight these flus that are out there. This I would say is ok to get the vaccines because the Iináájí [Life Way] ceremony tells about the Gila Monster who made the first herb and it said there will be different types of things that will be coming but I will be here to guide you and to show you which herb to use.”* The TKHs expressed assurance in connecting with the creation stories as a positive way to think about and frame COVID-19, and each expressed confidence in the Diné people’s resourcefulness in finding traditional remedies. Another TKH shared the following creation story:

*“When they killed the Yéi’ii Tsoh [monster giant] and when they were taking its head, it spoke to them and said I shall return sometime in the future as an illness. In our ceremony, we make references to this illness as an invisible spirit that predicted its return not as Yéi’ii Tsoh but as various illnesses to inflict pain upon the Holy Surface Earth People (Diné). Sickness will come upon us to remind us of certain things that we are not doing right such as forgetting our language, ceremonies, and our traditional way of life. That is the way the coming of the COVID-19 seems to be.”*

It became easier for Diné people to understand COVID-19 once it was identified with a name using Diné terms. One TKH stated, *“Dikos Ntsaaígíí Náhást’éíts’áadah…it [COVID-19] was named but through ceremonial system and through Navajo Diné life way, Diné cultural system, and Diné cultural concept, we always refer to something that is intrusive as naayéé.”* Another TKH shared, *“The Diné Hataałii Association referred to it as the effects of the departed enemy.”* Table 2 includes a list of how TKHs identified COVID-19.

Barriers for traditional lens towards COVID-19—On the other hand, there were also varying viewpoints and concerns expressed by the TKHs on the lack of research or explanations offered from the Diné perspective and they specifically questioned the origin of the vaccine. It was difficult for TKHs to understand details related to the COVID-19 vaccines if it was not approached or explained from within a traditional lens. An example of the lack of research from the Diné perspective, is further explained by a TKH who shared, *“We don’t research, we don’t find out what type of medicine this Moderna, Pfizer is—where is it from? No one is asking.…We were told the vaccines were to make us immune to the coronavirus, that’s it. We all kind of went with what the providers at IHS told us.”* Another TKH was worried about side effects from the vaccines, *“Whatever medicine was made according to the Caucasians, made by five fingered beings, well that probably in some way has an aura. It has a side effect”.*

#### 3.1.2. Education

Promoters for education—During the pandemic, educating the community on the NN was crucial to slow the spread of COVID-19. The patterns for promoters for education were understanding both the western and Diné explanations about the virus. One TKH expressed the importance of education, *“Learning the disease process and educating the Diné people about it because they don’t understand the process,”* and emphasized the need for community comprehension from a western perspective. Another TKH shared that young people also needed education so they have greater traditional comprehension, *“Our youth lack the knowledge of our traditional medicinal application. If our elders and traditional medicine men can be participants and share their knowledge with the public health and their knowledge included in curriculum it might be less complicated. Also being mindful of where we are presently at and what we still have as far as our traditional knowledge is concerned, it would be beneficial to our people.”* In addition, since elders were a vulnerable population, it became a priority to promote traditional comprehension (to safeguard elders by educating them) by translating as much educational and preventative information about the virus. As one traditional healer highlighted, *“Some of the medicine women and men spoke on KTNN when the pandemic started by telling the people how to take care of themselves, how they should pray, and to believe in their ceremonies.”* Although there is a language barrier, traditional healers were able to help educate, inform and translate as best as they could for those whose first language was Diné.

Barriers for education—The patterns that created barriers to educating the Diné people were limiting or restricting education efforts to only include western knowledge. Other barriers cited were the limited opportunities to promote education in the Diné language, and the lack of Diné-based educational materials. One TKH stated, *“It was just information that we gathered either from social media or from the IHS. So that is, this [COVID-19] is what it is, this is what it does—this was it. We didn’t really educate, no one really went down to the story.” It was difficult to locate resources to help aid in this effort because there were strict restrictions for the communities. Social distancing was in place, and it was hard to produce and access educational materials. A TKH highlighted,* “*This information needs to be reported in Diné language, you know, have knowledge in what these vaccinations have [ingredients]. Our grandparents, they don’t know about this kind of information, they only hear what is shared with them. That is how it is.”* To eliminate barriers, the TKHs recommended use of Diné bizaad [Navajo language] in public health messages and the integration of culturally relevant words in education about the virus and vaccines.

#### 3.1.3. Vaccine

Promoters for vaccine—When availability of COVID-19 vaccines were announced, there was a lot of positive feedback and a return of hope to the Diné people. Some patterns of promotion (for vaccine uptake) included the increase in vaccine education, sharing information on the history of vaccinations, and the influence younger generations had for encouraging their elders to get vaccinated. Vaccination uptake could be promoted through education and explanation using a traditional lens. One TKH holder shared, *“Medicine people need to come out and talk to our people to explain the way the virus has come and is affecting us. A vaccine is available and will help. If they are told through cultural ways, they will get vaccinated.”* Another TKH stated, *“Since a vaccine has become available, we should not be afraid to get vaccinated. Based on the Diné teaching, is it okay to get the vaccine? Yes, it is okay… With prayers and chants, they are effective”*.

One TKH referenced the historical context of vaccines as a reason to get vaccinated, *“Therefore, you look back again and again to various pandemics that our people survived thus far. In those pandemics and epidemics…Indian Health Services and Public Health Services [provided] medicine and vaccines. So, we continue to utilize those psychotropic medicine and those Western medicine because sometimes it’s okay to utilize it.”* The ability to combine knowledge and information from both western and traditional perspectives created a fresh new outlook on vaccinations and how being vaccinated would benefit not only a single individual, but the nation as a whole.

Overall, the TKHs supported the use of vaccinations as a life saving measure and most indicated they were vaccinated. One TKH stated, *“Although the virus is still here, we have the vaccine to help us. Many of our medicine people have been vaccinated and they like it and still depend on their traditional prayers and ceremonies to be strong.”* Many TKHs expressed how they were encouraged to get vaccinated for their own safety, and for the safety of their extended families, and grandchildren and to safeguard themselves so they could be around to watch them grow. TKHs also describe their roles in encouraging vaccination. One TKH stated, *“It was viewed as a remedy to the virus that has become available. Now, these hataałii ask their patients if they have been vaccinated.”* TKHs are given the utmost respect within the Diné community, and their opinions are highly regarded. Their decisions to support the vaccine are cited as having a positive impact on community vaccine uptake and was one way to ensure traditional ceremonies could safely continue as a means to help benefit their people.

Barriers for vaccine—The barrier patterns for vaccination included feeling there was no choice and feeling hesitant. One TKH shared, *“It is like we don’t have a choice. You either get vaccinated or risk being exposed and getting the virus. So, we don’t have a choice. It is unavoidable.”* Reasons people were hesitant included preference to use their own Diné medicine and lack of a full intercultural explanation or discussions on the risks and benefits of the vaccine. One TKH explained this hesitancy further and indicated getting vaccinated was *“a divided issue as some will still say, ‘no, I have my own medicine to protect me’ and others will say, ‘it is good, and it was made to protect us.*” In addition, Diné people were *“apprehensive about it because they think it’s the actual virus being injected,”* and felt there, *“should have been discussions about how the vaccine was created before people agreed to be vaccinated.”* Another reason for hesitancy was hearing *“stories of certain people getting vaccinated, and they encountered complications.”*

### 3.2. Harmony and Relationships

#### 3.2.1. K’é

Promoters for k’é—K’é is described as the relationships between family, clans, and kinship systems. In Diné culture, clans form an individual’s identity and help establish connection within Diné society through membership in four maternal and paternal clan groupings. The patterns of promotion under the sub-theme k’é included ensuring family and medicine people were safe and having strong homes. Some of the medicine people indicated that precautions were taken to protect entire families from COVID-19 transmission, and it became a community effort to ensure safety of family and the larger extended NN community. One TKH indicated, “*So some of the hataałii [medicine men and women] and patients require that there be hand sanitizer at the doorway. The community members and patients are taking care of the hataałii and the hataałii are also taking care of themselves.”* Most TKHs expressed they offered ceremonies for others, but due to the lockdown and stay-at-home policies implemented by the NN government some TKHs may have only been able to offer services to those who were within their immediate family. Another TKH mentioned, *“But, you know, we protected our own families, we protected our own relatives, our immediate relatives. But we didn’t attempt to help anyone outside our families.”*

Strong homes included households that had access to resources (i.e., transportation, running water, electricity) and maintained mental, physical, and spiritual practices. Having a strong home ensured family members stayed safe and protected, particularly when they used cultural teachings and ceremonies. One TKH shared, *“That [COVID-19] really impacted…our belief, our ceremonial ways. In our households, we burned our cedar and performed protection ceremonies.”* Another TKH commented, *“Some homes are strong, others are weak. Strong homes haul wood. Weak ones were greatly affected by the virus mentally. Some stayed strong despite the hardship.”*

Barriers for k’é—The barrier patterns were identified as things that hindered k’é (family relations) and include death, the modified burial procedures during the pandemic, and inadequate access to infection mitigation resources (i.e., water, PPE) that increased infection risks for TKHs. The TKHs commented that people who died during the pandemic were often alone and removed from family due to safety, but it was a different experience than what should take place from a Diné perspective. One TKH shared, *“The whole process of burying their loved ones was missing the reception and gathering to have relatives come to support them. There was no closure, and it caused a painful impact mentally and physically.”* During the pandemic people on the NN often found it challenging to get PPE and necessities such as food and water, and those who were most vulnerable were elders. One TKH shared, *“We couldn’t find things to help our elders due to the shortage.”* In Diné society, elders are revered and often taken care of but the pandemic and lock down policies made it challenging to reach this specific population. Another barrier that went against teachings for k’é were how some people did not take precautions to protect their medicine people. Another TKH stated, “*The families of deceased medicine people blame various people for being responsible for what had happened. Our hataałii were fine and they [other people] affected them. They [other people] lied and some came back with the virus from Utah and Phoenix and affected and caused the death of our grandfather and grandmother.”*

#### 3.2.2. Balance

Promoters for balance—Balance refers to actions or events that support or hinders one’s thinking, overall wellbeing, or their relationship with the environment. The patterns for promoters of balance under the original theme of harmony and relationships included taking care of oneself and making offerings to the environment. Some TKHs stated that to take care of family and community members it was essential they find balance in their own lives by following measures for self-care. One TKH commented, “*But, you know, as a practitioner, as a hataałii, we’re supposed to do a lot of self-care. We’re supposed to do a lot of [self-care], that should be number one because, in order to provide, in order to heal a person, in order to do ceremonies, you have to be well.”* In Diné teachings, to maintain life balance, including spiritual balance, one must practice reciprocity and not just take but must also give. This is often done by offering sacred stones, corn pollen, or cornmeal to the Diné Holy People of the environment. One TKH explained, *“We as Natives don’t just take things from the earth and sky to make a profit or to explore things.”* Another TKH shared, *“As far as Natives, especially Diné, we made an offering for all things that we take from our environment…we make offerings to get resources just enough to use.”*

Barriers for balance—The barrier patterns that prevented balance within category of harmony and relationships included not making offerings and influencers of global warming. The THKs expressed that if reciprocity is not practiced between humans and their environment it creates a destructive pattern that leads to imbalance within all life forms. One TKH explained, *“I have always said if you take things from nature or if you take too much or add something to it then it becomes destructive. If you take it out of its natural state by adding certain chemicals or deleting certain things for the purpose of getting rich, there will be dangerous consequences and can become deadly and it will become destructive and can easily lead to people dying from it.”* On a similar note, another TKH shared, *“A lot of people used to come to these ceremonies and put down offerings for their family, Mother Earth, the universe, and the four cardinal directions. Somewhere along the way we forgot that. At the creation of the earth, the holy people thought, talked, and placed everything in their proper place. They started moving and will continue to move and we will walk and sit on Mother Earth. Through these teachings, we are supposed to make offerings.”* In concert with not providing offerings, the TKHs expressed that humans are not in harmony with the environment, and this is influencing global warming. One TKH shared, *“From my point of view, that is where global warming came about because we were supposed to make mineral stone and corn pollen offerings back to the Holy Deities and that belief has gone away. This has shifted back to us not as a punishment but rather as a wake-up call reminding us of the traditional things that we need to do.”* Another TKH stated that an imbalance in the environment will cause unforeseen consequences, *“According to the ceremonial stories, there was a time when naayéé’ [monsters] came into existence and there became an imbalance in our environment. Some of our elders tell of a story where the earth and the sky disagreed, and something happened. If that imbalance happens again with nature, there will be some consequences that will occur.”*

#### 3.2.3. Food

Promoters for food—Perspectives on food had an essential role during the COVID-19 pandemic and brought to the forefront the importance of Diné traditional food for sustenance and overcoming the challenges related to health outcomes. The patterns for promoters of food that support the parent category of harmony and relationships included the belief system that animals and food are medicine and people should follow food protocols. In Diné belief systems, the food cycle is connected to the wellbeing of humans due to the physical and spiritual connections, so food is essentially viewed as medicine. This connection is strengthened when the food system is respected and valued in its most natural state. One TKH shared, *“As Diné people, we were given cornfields, natural fruits and natural vegetation and we grew our own food. We had our own livestock and wildlife that we used. When these livestock and animals partook of the vegetation that were not sprayed with fertilizer and chemicals, they were healthy, and the vegetation are considered medicine in our way of life. When these animals ate this vegetation and we as Diné people partook of these animals, it was medicine all the way around.”* Diné teachings also expand on food protocols that must be followed to live a balanced life. This is shared by the teachings of one of the TKHs, *“The only types of food that we are supposed to eat were healthy food [like] corn, squash, and all these other vegetables that are out there and the sheep of course. The sheep is one of our main foods and my grandfather used to tell me whenever you’re physically, mentally, spiritually, and emotionally down, always turn to a sheep/lamb. When we butcher, we use that in our big ceremonies where they do the mixture of food and pray for it. Just drinking the broth from it will calm you down and will give you the strength to move forward.”*

Barriers for food—The barrier patterns for food that caused disharmony included consuming processed food and animals that were considered enemies. Diné believe when the food cycle is disrupted, it translates to poor health outcomes for humans and the natural world. The medicine people shared how contemporary food systems and food choices are creating undue harm because Diné people have strayed from their traditional food sources. Food has spiritual significance in one’s life and if it is not adhered to it disrupts the sacred relationship between animals and humans. One TKH expressed, “*But nowadays there are many processed foods which have been treated with chemicals and all kinds of things that are added and we as Diné have never eaten those kinds of food. But once the non-Native people have arrived, we begin eating those kinds of food.”* In Diné teachings, there are certain animals that must never be eaten due to their sacred properties that could disrupt health and wellbeing for humans. One TKH shared what types of food should not be consumed, *“There are certain foods like dogs, reptiles, and bats that we are not supposed to eat.”* Other food that should not be consumed include seafood, as shared by another TKH, *“There are other kinds of food that have been gotten from the Ocean where they have been exposed to tornadoes and lightning and that can affect us if we eat them. It is probably our own fault if we eat them.”*

### 3.3. Spirituality

#### 3.3.1. Traditional Knowledge

Promoters for traditional knowledge—The patterns for promoters of traditional knowledge, under the parent category of spirituality, were the sharing of creation stories by medicine. Diné creation stories and the Hero Twin stories have long influenced the lifestyle of the Diné people. The stories describe how twin brothers, Monster Slayer and Born for Water, slayed evil monsters but left several naayéé’ alive. In Diné culture, traditional stories indicate how people must live in harmony and balance to avoid unbalance and disharmony. One TKH indicated, *“Laws were made to control all things that are not good or evil.”* Teachings and ceremonies that were passed to keep harm at bay are continuing. When COVID-19 suddenly and unexpectedly hit the NN, it was called a naayéé’ [36]. TKHs related how traditional knowledge could help Diné people understand the virus. One TKH described it as a *“monster and is harmful, destructive and can deteriorate [the] mind, body, soul, and spirit.”* Another TKH shared, *“This naayéé’ is dangerous and can kill. Reflecting on our traditional stories, there were certain naayéé’ like the Yéi’ii Tsoh [monster giant] and ‘the one that kills with its eyes.’ What did it use to kill people? How did it kill people? What was its weapon? If this can be figured out about the virus, then we would know how to treat this naayéé’. This is how our medicine people would look at it, and in their ceremonial setting they don’t say COVID-19 but address it as naayéé’”.*

Barriers for traditional knowledge—The COVID-19 pandemic is a modern-day monster that confronts the Diné people and has significantly impacted the NN. The patterns for barriers of traditional knowledge include violations of traditional laws. For example, the acknowledgment and the controversy regarding the naming of COVID-19, using the Diné language was cited as a pattern barrier to honoring spiritual laws and practices. Many Diné people believe that words are powerful and spoken words or evil thoughts can bring illness. One TKH expressed how *“it was our [Diné people] fault”* for giving it a name, and *“using that name, it [COVID-19] grew into a living thing and spread among the Navajo,”* taking loved ones and many relatives. Another TKH stated, *“Our tongue is sacred, and we should not use it to assign a name to an illness, especially within the four Sacred Mountains.”* A TKH expressed frustration that a name was given to COVID-19 without insight from TKHs, “*When COVID spread on Navajo, the leaders gave the virus a name. This is how the Diné medicine people didn’t have a say so. We were not asked by the leaders, ‘What are your thoughts on this pandemic? What is your input on the giving the virus a name?’ The virus was given a name, and that is what happened”.*

#### 3.3.2. Traditional Practices

Promoters for traditional practices—The patterns for promoters of traditional practices included enduring songs/prayers and ceremonies. The NN has relied on western medicine but has never strayed away from traditional teachings and ceremonies, especially during difficult times like the COVID-19 pandemic. One TKH stated, *“I believe people became closer to the belief systems. They only gained strength through prayers, chants, herbal medicine, and ceremonial processes. In that way, people became stronger.”* When the NN was under strict regulations, large gatherings for ceremonies were prohibited. However, several TKHs continued their efforts to help their community. *“I did not stop doing ceremonies, but I controlled the number of people, followed the requirements of wearing a mask, using hand sanitizer, even wearing gloves and taking temperatures. I still follow these protocols as I conduct my ceremonies and require only five people or less,”* stated one TKH. Many TKHs expressed that the virus would leave the NN because of the continuous prayers, offerings, and ceremonies conducted. As one TKH mentioned, *“I feel that we as medicine people helped through the ceremonies, and it is not solely the doings of the scientists. The crystal gazing, protection prayers, and other ceremonies that we used helped.”* For traditional Diné, these cultural beliefs promote personal and community health and work to restore balance and harmony between the spiritual and physical worlds.

Barriers for traditional practices—Patterns for barriers of traditional practices, or things that contradicted or restricted spiritual and cultural teachings, included not feeling prepared for the pandemic, thinking negative thoughts, and dealing with an unpredictable virus. One TKH explained, *“It came so fast that we as medicine men were not prepared for it because we were told you do not prepare for anything negative you just take it as it comes.”* One TKH also noted how difficult it was to pray for people because the *“virus appears to protect itself and is unpredictable.”* When the NN was hit with the peak cases and death due to COVID-19, Diné people were reminded to not resort to negative thinking. One TKH urgently stressed, *“We do not know what is happening and where we are going. Maybe it [COVID-19] might eliminate all of us. This is the kind of thought you have at times. Even though our elders say you are not supposed to have these kinds of negative thoughts, after witnessing the sickness and eventual death of some of your close relatives, you cannot help thinking those thoughts.”* Many Diné elders and TKHs were taken by the virus and, along with it, the knowledge, traditional teachings, and language they held. A TKH cautiously expressed, *“You cannot discuss such a deadly energy/item without some form of spiritual protection. I know of some medicine people who tried dealing with the pandemic and have been affected and lost their life”.*

#### 3.3.3. Traditional Medicine

Promoters for traditional medicine—Diné traditional medicine has been utilized since the existence of the Diné people. Diné Holy People (Diyin Diné’e) passed sacred ceremonies, chants, and herbal medicine to specific Diné people such as the hataałii who can communicate with them. The patterns for promoters of traditional medicine were the application of knowledge of herbs and prayers that were used for protection and strength. Several TKHs indicated that to keep strong mentally and have good health, one must use herbs, chants, and prayers. Some of the herbs mentioned included, *“dark herbs, purification herbs, spider herbs, bitter herbs, sage and many others.” “Make sure you have some form of protection. Maybe an arrowhead, protection prayers, protection chant, the Bible, the sacrament peyote, or another form of protection with yourself before you go out and talk about the virus because it is dangerous,”* one TKH said. Traditional ceremonies are vital in the Diné culture. There are various types of ceremonies that depend on what kind of help the patient is seeking. One TKH explains why he did not stop performing his duties as a medicine man during the pandemic: *“the ceremonial objects that you have are alive and wise, and it is guiding you and will protect you. Mentally you will think positively, and mentally you will be strong because people look up to you. You are there to protect them and be there as their shield. If you stop, you would be closing the door and saying you don’t need the help of the Holy people. That was one of the big reasons why I didn’t stop my ceremonies”.*

### 3.4. Respect for Self and Discipline

#### 3.4.1. Diné Identity

Promoters for Diné identity—Diné identity refers to the values, beliefs, and principles (e.g., history, teachings, practices, language, kinship) of the Diné people. The patterns for promoters of Diné identity (respect for self and others, and discipline) included taking care of oneself, respecting Mother Earth, and understanding that the Holy people watch and know. These teachings remind the Diné to remain self-aware and to carry themselves per the teachings of their ancestors. TKHs explained how in a ceremonial setting, there are protocols set in place that teach Diné people restriction and reverence. One TKH stated, *“Living as a Diné, you follow these teachings and live your life in that manner every day.”* Many Diné people implemented cultural teachings instilled from their upbringing during the pandemic for themselves and their families. One TKH shared, *“The Diné concept of self-care is self-protection, remembering your life and being reverend, which is viewed as a wellness care, and you practice that in your life. This way of living should be for yourself, your spouse, and children.”* Diné TKHs believe *“a life of being reverend is all encompassing”*, as it *“follows a pattern of seeking help, looking to help, being reverend, being hopeful, depending on your prayers, songs and belief, getting well and continuing with your life in perfect mental and physical state of being.”* TKHs also voiced the importance of respecting Mother Earth to strengthen the connection to the land and environment. One TKH stated, *“This supports what our elders have said about the results of not respecting the air and the illness that will follow. For that reason, our elders have said always be aware of your environment and consider them sacred like plants, insects, birds, and the waters.”* In relation to respecting Mother Earth, TKHs believe, *“The Holy People are watching daily and nightly and they know what is going on.”* The Diné belief system views Diné people as “Holy Surface Earth people” and this concept demonstrates reverence to the Holy people through acts of following traditional protocols and instilling respect and self-care practices day and night.

Barriers for Diné identity—Barrier patterns that interfered with maintaining Diné identity, Diné values and principles included not respecting medicine bundles, food, fire, and COVID-19 protocols. When ceremonial protocols are not followed properly, Diné people often believe it causes a disruption to the environment. One TKH shared, *“We don’t consider anything with respect anymore. We are also performing our ceremonies inappropriately. These stars, and these current events, and the seasonal changes, we are no longer in sync with these natural cycles, perhaps this is the reason for what we are facing today…Then our boundaries, our ceremonies, are also the same. We also don’t respect/acknowledge our sacred mountain bundles; we don’t care for our bundles.”* TKHs believed not respecting medicine bundles caused a disruption—the spread of COVID-19. TKHs also voiced the importance of acknowledging food and fire as sacred beings. One TKH stated, *“When my grandmother used to prepare food, she talked/prayed to the fire and acknowledged by feeding food to the fire. My grandmother prayed for the food before we consumed it, she prayed for mental health and physical health for her children, as healthy persons, this is how she took small pieces of food and offered it back to the fire.”* Traditional practitioners often believe when Diné people obey traditional food practices, it maintains a relationship with Mother Earth. In addition to not following traditional protocols, TKHs observed many Diné people not following NN curfew protocols. One TKH shared, *“They would still go through the back roads to town even though they were told not to go places. There were roadblocks, like we had a roadblock in Hogback, but a lot of people still went through the back road to get to Farmington. During those times, a lot of the virus was brought back into the Navajo Nation. Some did not respect authority”.*

#### 3.4.2. Discipline

Promoters for discipline—Discipline played an essential role for TKHs and Diné tribal members when following COVID-19 protocols. The patterns for promoters of discipline included self-discipline and community-discipline. During the early months of the pandemic, the NN tribal government implemented protocols to ensure Diné tribal members enforced a mask mandate, social distancing, limited travel, and large social gatherings. TKHs believe cultural teachings and respect for others strengthened the need to obey NN COVID-19 protocols. One TKH stated, *“In addition to physical cleanliness, physical discipline was also stressed. We were told to exercise by running and these were the teachings that we grew up with. So that discipline was already instilled in us when we were growing up. When the pandemic came among us, we already had the discipline which we taught our children and our relatives.”* The Diné people often believe discipline is not only practiced outside the home but inside the home as well. Many tribal members honored k’é when being mindful of travel and others to instill respect for traditional principles and teachings. One TKH explained, *“As for your activities (where you go, how far you go, why you go) you should have that discipline already in place. So, to follow that and say I respect you, you should stay home and should not go to other people’s homes and risk those people. So, it is more of staying home, taking care of your home, taking care of yourself and using your faith, belief and whatever you have to give you the strength and hope for a better tomorrow.”* As a community, social gatherings and traditional practices had to be limited to abide by the set guidelines. A TKH mentioned, *“Navajo practitioners who are a part of the Diné Hataałii Association came up with a proclamation that spelled out how we should conduct our ceremonies and it was sent out to all practitioners. It gave us an opportunity to ponder our ceremonies and consider how we can continue on with them in a respectful manner and show reverence to them”.*

#### 3.4.3. Personal Health

Barriers for personal health—TKHs expressed how the personal health of family members and community were “disturbed” by the impacts of COVID-19. The patterns for barriers of personal health included detrimental impact to both physical and mental health. Many TKHs stressed the mental impacts the pandemic had on Diné people and voiced, *“It brought in a lot of emotional stress, anxiety and depression.”* One TKH explained the need for traditional practices to be held via telephone during the early stages of the pandemic. He shared community members who were affected by the virus expressed themselves in tears via telephone explaining they were “highly stressed” by the “pressure of life.” Apart from the mental impacts, TKHs observed the long-term effects of COVID-19. One TKHs stated, *“It has long term effects. I experienced that due to damage that it has done to my lungs. I have shortness of breath and I get fatigued really easily.”* The Diné people and TKHs were not prepared with the health impacts caused by COVID-19 or the dangers it created for populations at risk. One TKH stated, *“There were people in the age range of 15 to 30 who were not mindful and were not careful and did not follow guidelines. These were the most vulnerable people”.*

## 4. Discussion

### 4.1. Lessons Learned from Traditional Knowledge Holders

The knowledge and stories shared by Diné TKHs offer invaluable advice for understanding the COVID-19 pandemic and vaccine hesitancy, and for creating future mitigating factors to help offset public health challenges in preparing for future pandemics. The narratives also identify several lessons learned from the experiences of TKHs that can be integrated within the Diné perspectives of harmony/relationships, spirituality, and respect for self/discipline. In particular, the lived experiences of TKHs during the pandemic showed the strength and resilience of Diné culture and medicine people. This forte was demonstrated as they served as role models, shared their cultural knowledge and continued ceremony by adapting their practices to ensure safety was prioritized through smaller gatherings, telephone ceremonial consultation rather than in-person, and enforcing the use of masks and hand sanitizers.

#### 4.1.1. COVID-19 and Vaccine Hesitancy

Although the NN has high COVID-19 vaccination rates, the NNDOH continues to safeguard their community by stressing the importance of vaccination, which includes reaching out to those who remain vaccine hesitant. According to Denetclaw et al. [37], by May 2021 over 85% of Diné people on the NN over 16 years old received the first dose of the COVID-19 vaccinations. Rates of COVID-19 vaccinations were inversely associated with COVID-19 cases and deaths in the NN [37]. As of February 2023, 71.2% of Diné people from five to 65 years old and older completed their vaccination, and 24.2% stayed up to date by receiving the booster shot [38]. The Diné elderly population 65 years old and older has the highest percentage of completing the COVID-19 vaccination at 91.2% and Diné children five to 11 years old have the lowest rate at 53.4% [38].

One lesson learned from TKHs is the importance of framing educating on COVID-19 and vaccines within a traditional lens because many Diné refer to creation stories and teachings to deal with life adversities. For example, the teaching about Gila Monster provides reassurance on how western medicine and vaccines are useful in modern times and can be an extension of Diné medicine and herbs. The creation story about Yéi’ii Tsoh helps remind the Diné people to use their cultural strengths like ceremonies, prayers, and language to get through the hardships and trauma associated with the pandemic. Careful use of language, such as naming a virus (an enemy) requires cultural consideration due to the significance, meaning and the impact language has on outcomes. Diné view all living beings, even viruses, with utmost respect. In the future, seeking input from TKHs is strongly recommended before decisions about language and naming of living entities such as viruses are decided. Consultation with TKHs ensures actions are culturally respectful, safe, and appropriate. 

To help address vaccine hesitancy more transparency will be necessary and should include details about how the vaccines were made. In addition, it is recommended to integrate a traditional Diné lens to describe the research risks and benefits in detail to create greater opportunity to accurately convey information using Diné teachings and terminology. The TKHs in this study served as public health ambassadors, and their decision to be vaccinated promoted greater acceptance of the vaccine among their patients, while reaffirming continuation of safe ceremonial practices. TKHs served as leaders and agents of change as they safeguarded their communities. Consistent with the findings on the NN, Haroz et al. [39] confirmed elders and traditional healers from other AI communities were influential in supporting vaccine acceptability and promoting vaccinations. The TKHs play a tremendous role as educators, leaders, and ceremonial practitioners and are key players in helping Diné people understand health related impacts of COVID-19 and vaccines.

#### 4.1.2. Harmony and Relationships

TKHs had a huge role in helping community members stay spiritually strong and to remind people to continue to follow traditional and western protocols to protect each other and relatives. Honoring and respecting the teachings of k’é is a lesson they emphasized, and these teachings were used to protect Diné people from COVID-19 and to increase vaccinations rates for the NN. Encouragement received from grandchildren was cited as the main reason why most TKHs in this study decided to get vaccinated and their agreement to be vaccinated supported mass vaccination efforts in the community. Diné kinship teachings and values are based on individual identity (clan relations) and connecting through kinship to the larger clanship system. K’é (clan kinship, family) relies upon concepts such as love, compassion, peace, kindness, and generosity [31] to maintain relationships. Teachings about k’é are central to maintaining the Diné culture. The Diné way of life is based on the connection and interrelations not only to K’é but also with Mother Earth, Father Sky (the universe), and the Holy People. The four domains critical to health and wellness include, self-identify, self-respect, protection to self, and resilience [40]. The Diné people have passed traditional knowledge through storytelling and ceremonies. Through storytelling, the Diné people have sustained cultural teachings, language, and social norms [41,42]. The knowledge of these traditions are crucial for the survival and protection of individual and collective health. Teachings of respectfully carrying this knowledge forward must take priority to preserve the traditional lifeways of the Diné people.

#### 4.1.3. Spirituality

Another message shared by TKH is the need to build a stronger infrastructure for health messengers in rural communities that integrate traditional perspectives about health, specifically information on illnesses and viruses. Mheidly and Fares [43] recommend using targeted health communication strategies for minority populations and specific races to address COVID-19 misinformation sharing. They recommend using role models of similar races to make messages more relevant for health awareness campaigns. Ignacio et al. [44] also reported on the importance of having trusted community leaders convey important public health messages and highlights how vaccinated leaders and elders (of the same racial/ethnic group) were effective in encouraging vaccination in their communities. The Diné community places great trust in TKHs and future public health initiatives would be strengthened if TKHs were consulted during the process of creating health educational materials, and while training health messengers who will educate the community. Previous AI studies integrated cultural knowledge into their materials and emphasized the greater effectiveness when including cultural beliefs, stories, and ceremonial knowledge to help shift behavior change for health promotion [25,45,46]. This process could address language barriers when health education materials are presented in Diné bizaad (Diné language) or with use of culturally relevant terms that consider the traditional worldview. The elderly would have greater understanding of materials and messages if they were taught using a Diné lens and incorporated stories and teachings from TKHs. The TKHs insights on stories and perspectives related to COVID-19 offered a plethora of knowledge regarding past vaccines and their roles in Diné society.

#### 4.1.4. Respect for Self and Discipline

The TKHs were adept in teaching and addressing language barriers, and also adapted to the need of using technology during the COVID-19 pandemic. TKHs also recognized and advocated for those who were not able to access technology and recommended approaches to reach those most isolated and remote with life-saving health education materials. The importance of radio stations was emphasized by some TKHs who used that platform, and each stressed the importance of finding new ways to reach rural communities. The TKHs also recognized the limitations of existing mental health infrastructure and recommended strengthening mental health infrastructure and services on NN. The TKHs addressed the mental health service gap by supporting the mental and emotional health of the Diné people using traditional knowledge and practices such as herbal medicine, oral history, messages of resilience, prayers, and ceremonies. Mental health support could be expanded through local organizations or other accessible settings such as online mental health support or help lines, and by training public health professionals on mental health referral and intervention strategies. In Canada, a study identified digital interventions used to support mental health and identified the following resources: telemedicine, websites with mental health courses or discussion forums, cell phones, text messaging, peer support offered virtually, and mobile mental health apps [47]. The experiences and advice offered by the TKHs provided cultural insights, on how this pandemic tested the Diné and served as an awakening to revisit cultural teachings and strengthen the ability to be self-reliant and use ancestral knowledge to overcome future challenges, including potential pandemics.

### 4.2. Limitations

A potential limitation of the study was the analysis of 12 of the 15 interviews; more insights, themes, or patterns may have been identified if all interviews were included. However, the research team agreed that 12 interviews were sufficient to reach data saturation. In addition, of the 12 interviewees, 11 were males; it is plausible that more interviews with female medicine people may have provided a stronger context for female TKH perspective. Another study limitation was the lack of demographic information on the TKHs to determine differences in age, type of employment, and education. Most of the interviews were conducted in the Diné language and the translation of words into English may have altered or diminished some of the meaning behind the interviews. However, the research team used the services of two Diné language experts who have extensive experience and confidence in translating and transcribing to minimize errors.

### 4.3. Implications for Public Health

By February 2021, the NN had reached 29,386 confirmed cases and 1127 confirmed deaths [48]. Two years later, as of February 2023, there were 80,808 cases and 2030 deaths on the NN [49]. The high number of COVID-19 cases and deaths indicate a need for a broad public health force, inclusive of the voice and input by TKHs. Involvement of TKHs through every stage of pandemic planning and mitigation efforts in AI communities is critical to ensure culturally safe and asset-based approaches are utilized. TKHs have an important role in helping community members understand the historical and spiritual perspective of catastrophic events like the COVID-19 pandemic. Future planning strategies must include TKHs on local and national advisory boards, which will ensure inclusion of traditional AI perspectives and culturally safe and respectful approaches to understand illnesses, viruses, and mitigation efforts from within a cultural context. Doing so would help a greater number of community members to understand health messages more quickly and help public health professionals establish trust and build rapport. TKHs could also offer a variety of strategies regarding healing, medicine, and mental health support for community members who are seeking additional support and guidance beyond western medicine. Integrating TKHs into western medicine contexts has resulted in patients feeling comfortable and empowered when treated with familiar traditional ceremonies [50].

Additionally, stronger communication efforts are needed between western-based health care providers and public health professionals and TKHs to ensure mixed messages are not hindering best practices of care and prevention. This step could be accomplished by developing a formative needs assessment to understand barriers and promoters that would help both TKHs and public health practitioners in planning efforts. In addition, funding should be set aside that would allow TKHs to be at the table in meetings and conferences, and through other platforms like the radio, social media, or websites, to have their voices heard and roles acknowledged. A stronger network connecting all TKHs would be beneficial to yield strategies that integrate perspectives to create stronger public health guidelines for communities.

Additionally, some TKHs commented that they were at the frontline just like western medical doctors, but they were not valued in the same way because they did not get extra compensation or support when needed. In Diné culture, TKHs have equal status as western doctors and may need enhanced support during public health emergencies (pandemics) to ensure they continue to stay safe while providing care and advice to many in the community that western doctors cannot reach. Another recommendation is to ensure that best practice efforts that include TKHs in the health care system continue to be maintained or strengthened. For example, if IHS clinics added TKHs to its teams it would be advisable to continue these services. The growing infrastructure and funding should continue to remain in place and community members should be informed of how mitigation involving TKH roles will continue. Additionally, more Diné public health messages and trained messengers could greatly benefit the Diné community. In one study, the DC public health program designed a 1-unit course to train students to be health messengers and proved to have many benefits [51]. The project was based on the concept of leveraging teachings of k’é, as students approached vaccine hesitant peers or family, as a relative, and offered health messages regarding COVID-19 and the vaccine.

### 4.4. Recommendations to Protect TKHs into the Future

Future efforts prioritizing support for TKHs to expand their roles and protect them as frontline health care workers are necessary. The number of authentic TKHs on NN is limited and the pandemic highlighted their potential vulnerability when providing services in the midst of a pandemic. One recommendation to protect and increase trained TKHs is to create training or apprenticeship opportunities allowing greater opportunity for those interested to become a TKH. In addition, the NN government must allocate funding and create space in various communities where TKHs can share their knowledge and cultural teachings. During the pandemic, the NN tribal government had an opportunity to allocate Coronavirus Aid, Relief and Economic Security (CARES) Act relief funding for the Diné Hataałii Association, but the funding request was not approved [26,52]. The study findings also indicated many young Diné may not be learning traditional knowledge or carrying forward Diné cultural practices. Additional funding to support the development of a dedicated cultural center for Diné young people to explore a pathway to become a future TKH is an essential component for keeping Diné traditional culture and ceremonial practices strong.

### 4.5. Future Research

More research is needed to build upon this current work to further understand the roles of TKHs and how their knowledge and expertise can be incorporated into future pandemic or public health interventions. The first step is to conduct a formative assessment to understand the social and cultural needs of TKHs during the pandemic and how they want to be included in pandemic planning or mitigation efforts within the NN or other governing bodies. Future interventions could also utilize TKH support and knowledge to build culturally based COVID-19 curriculum for health care providers and public health professionals. Adding their voice into the curriculum would enable community members to have greater trust in public health messages that also leverage teachings of k’é, Diné language, and traditional knowledge in public health messaging efforts.

Digital stories have been used for public health promotion and education and align with AI storytelling as a means to share teachings or lessons [53]. Development of co-created (inviting TKHs to collaborate) tailored, culturally aligned digital stories on infectious diseases such as COVID-19 and vaccines would be great approaches for health promotion in AI communities. Health message materials inclusive of TKHs perspectives can also be pilot tested to assess effectiveness in health communication and prevention activities. More complex topics such as the mechanisms behind the COVID-19 vaccine, using messenger RNA, will require adequate time to integrate western and cultural explanations (culture-based teachings), allowing for deeper and more thorough understanding of complex material.

## 5. Conclusions

The COVID-19 pandemic may be viewed as a standalone event caused by the SARS-CoV-2 virus, however the Diné TKHs viewed it as an interconnected imbalance of the natural world that impacted humans as a result of not adhering to Diné teachings and protocols. The TKHs offered new insight into the pandemic that has not been previously explored from the Diné perspective. This unique study gives greater insights into the barriers and promoters provided by TKHs, thereby allowing their voices to be included, prioritized, and elevated in future pandemic planning. The TKHs shared their perspectives about COVID-19, the vaccines, Diné traditional concerns about naming the COVID-19 virus, and the importance of co-created cultural revitalization strategies to address health outcomes related to the pandemic. The study findings demonstrate how knowledge and experiences of TKHs are crucial to support individual health, collective health, and to promote teachings of k’é, the foundation of Diné culture. The role of TKHs must be acknowledged by funding support for their services, training opportunities for those who want to become a TKH, and development of policies within the NN government that recognize the essential and critical importance of the roles TKHs have in the collective health of the NN. The important insights gleaned from the valuable interviews with Diné TKHs enlighten the public with perspectives from the Diné worldview, and ways to improve research, health care delivery, and public health interventions as we strive to achieve health equity in AI communities.

## Figures and Tables

**Table 1:** Themes and Patterns for the COVID-19 Parent Theme (n = 12) Category.

Parent Theme		Themes	Patterns
COVID-19	Promoters	Traditional Lens towards COVID-19	Creation stories; identified COVID-19 with Diné terms
		Education	Traditional comprehension; western comprehension
		Vaccine	Grandkids influenced TKHs to get vaccines; previous experiences with vaccines (flu); reasons for vaccine; pro-vaccine; vaccine education; vaccine is okay to use and should be required
	Barriers	Traditional Lens towards COVID-19	No research from Diné perspective on vaccine; what is the origin of vaccine (fear of vaccines)
		Education	Few got on the radio to educate; only used western knowledge to educate; more Diné doctors are needed; Diné-based educational materials are needed
		Vaccine	Did not have a choice; hesitancy

**Table 2:** How TKHs identified COVID-19.

Words to Describe COVID-19	
Bił Ni’níchilí (the one not seen) The enemy The effects of the departed enemy Naayéé’(monster)	An illness that is not good The particles of something that is not good

## Data Availability

The data presented are not readily available because the data belong to the Navajo Nation, according to the Navajo Research Act and longstanding IRB policy, so any data sharing would have to be specifically approved by them, not by the authors. The dataset is small and includes details that could potentially reveal the identity of individual subjects. Requests to access the data should be directed to Nicolette Teufel-Shone at nicky.teufel@nau.edu.
